# 

**Published:** 1900-08-11

**Authors:** 


					THE HOSPITAL. "The standard of highest pntityj*?Lancet. August 11th, 1900
CADBURY'S i#|} ?? CADBURY'S
COCOA ^ m COCOA
AB80LUTSLY PUU, NO DRUGS OR ALKALIES USED,
?1
?J MM ~-.S -- S.7!/homass Hospital7^*-
a sc?* Western Infirm aw ~ " "" 'NDMOLKauo nor mcH # -a . JPp
No. 724. Vol. XXVIII. gjggjg] Saturday,
August 11, 1900.
[SubscriptionTeniiB.j pRICE TWOPENCE.

				

